# Nanoparticles Mimicking Viral Cell Recognition Strategies Are Superior Transporters into Mesangial Cells

**DOI:** 10.1002/advs.201903204

**Published:** 2020-04-22

**Authors:** Sara Maslanka Figueroa, Daniel Fleischmann, Sebastian Beck, Philipp Tauber, Ralph Witzgall, Frank Schweda, Achim Goepferich

**Affiliations:** ^1^ Department of Pharmaceutical Technology University of Regensburg Regensburg 93053 Germany; ^2^ Department of Physiology II University of Regensburg Regensburg 93053 Germany; ^3^ Department of Molecular and Cellular Anatomy University of Regensburg Regensburg 93053 Germany

**Keywords:** mesangial cells, multivalent nanoparticles, renal targeting, stepwise cell recognition, virus‐mimetic

## Abstract

Poor drug availability in the tissue of interest is a frequent cause of therapy failure. While nanotechnology has developed a plethora of nanocarriers for drug transport, their ability to unequivocally identify cells of interest remains moderate. Viruses are the ideal nanosized carriers as they are able to address their embedded nucleic acids with high specificity to their host cells. Here, it is reported that particles endowed with a virus‐like ability to identify cells by three consecutive checks have a superior ability to recognize mesangial cells (MCs) in vivo compared to conventional nanoparticles. Mimicking the initial viral attachment followed by a stepwise target cell recognition process leads to a 5‐ to 15‐fold higher accumulation in the kidney mesangium and extensive cell uptake compared to particles lacking one or both of the viral traits. These results highlight the relevance that the viral cell identification process has on specificity and its application on the targeting strategies of nanomaterials. More so, these findings pave the way for transporting drugs into the mesangium, a tissue that is pivotal in the development of diabetic nephropathy and for which currently no efficient pharmacotherapy exists.

## Graphical abstract

Nanoparticles (NPs) endowed with viral targeting strategies are presented. A triple‐check for cell identity is implemented by mimicking the initial viral cell attachment, followed by a stepwise enzyme‐activated recognition and internalization. Particles with these viral attributes are able to accumulate in mesangial cells, a therapeutic target of paramount importance due to their involvement in kidney diseases, such as diabetic nephropathy.

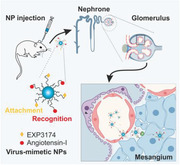

## 1 Introduction

Myriads of nanomaterials have been developed over recent years as carriers for drug therapy or diagnostics. To outfit them with the ability to identify target cells with sufficient specificity in vivo, ligands that bind to cellular receptors have been tethered to their surfaces.^[^ ^1^, ^2^ ^]^ However, simply following this old paradigm^[^ ^3^ ^]^ increased nanomaterial's avidities,^[^ ^4^ ^]^ but turned out to be insufficient for unequivocal cell identification.^[^ ^5^ ^]^ Not even nanoparticles (NPs) that present several different ligands for hetero‐multivalent binding^[^ ^6^ ^]^ are able to distinguish between different cells types. Viruses in contrast, are NPs with ultimate target cell specificity that make use of a consecutive multistep process for cell identification.^[^ ^7^, ^8^ ^]^ We recently showed that mimicking the sequential recognition strategy of influenza A virus, supplied nanomaterials with sufficient specificity to discern sharply between co‐cultured target and off‐target cells in vitro.^[^ ^9^ ^]^ However, a close‐up comparison with the way how viruses interact with cells made clear that the latter execute consecutive identification steps that had not been implemented into the former to date. They resemble “if‐then‐else” elements in computer programming^[^ ^10^ ^]^ that deserved more attention for the design of NPs. Especially the initial step of viral attachment to cell membranes, which does not result in particle uptake^[^ ^11^ ^]^ but increases virus particle density on the cell surface and aids receptor recruitment is missing in contemporary NP design strategies. More so, this initial adhesion to glycolipids and glycoproteins^[^ ^12^ ^]^ or specific receptors^[^ ^13^ ^]^ was found to be essential for viral infectivity.^[^ ^14^, ^15^ ^]^

It was, therefore, the goal of this study to investigate if nanomaterials outfitted with virus‐mimetic cell identification mechanisms could be addressed to a tissue of profound therapeutic interest in vivo and if they were superior to materials following conventional design criteria. We chose the kidney as a target organ, since it holds different compartments made up of various cell types with distinct functions. Among them, we selected mesangial cells (MCs) a highly relevant target of drug therapy due to their crucial role in the maintenance of the integrity of the glomerular filter^[^ ^16^ ^]^ and their severe impairment in several pathologies, such as mesangioproliferative glomerulonephritis and, more importantly, diabetic nephropathy (DN).^[^ ^17^, ^18^ ^]^ DN entails an enormous public health burden as it affects more than 50% of 425 million diabetic patients worldwide.^[^ ^19^ ^]^ It is the leading cause of end‐stage renal disease that can only be treated by dialysis or organ transplantation.^[^ ^20^ ^]^ Even though a number of drugs hold great promise to inhibit pathomechanisms, such as MC proliferation and extracellular matrix overproduction in kidney glomeruli,^[^ ^17^, ^21^ ^]^ they suffer from poor mesangial availability. DN therapy could, therefore, be revisited for a number of compounds if a nanoparticulate drug transporter was available that was selectively and sufficiently taken up by MCs in vivo. Even though early reports had shown that 70 ± 25 nm particles were most efficiently^[^ ^22^ ^]^ penetrating the glomerular capillaries’ 80–100 nm endothelial fenestrations,^[^ ^23^ ^]^ they are subject to mesangial clearance^[^ ^24^ ^]^ and hence inadequate for drug delivery purposes if they do not specifically recognize and enter MCs. Therefore, we regarded MCs as an ideal target of paramount medical relevance for investigating if there was a benefit of outfitting NPs with a viral mechanism of cell identification.

**Figure 1:**
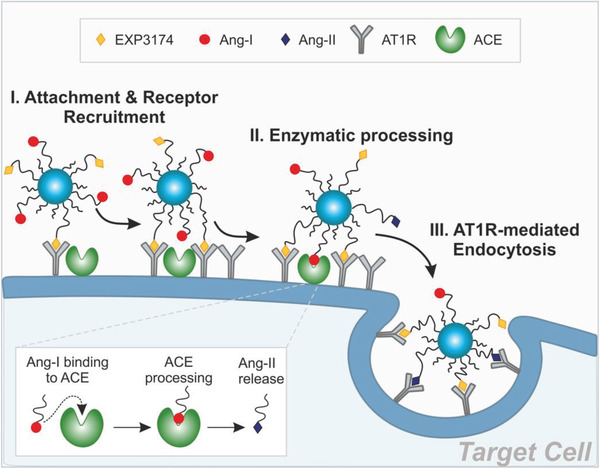
Virus‐mimetic attachment and target cell recognition. NPs carrying EXP3174 and Ang‐I on their corona (NPEXPAng‐I) attach to the cell membrane through EXP3174‐mediated AT1R‐binding. Specific recognition is triggered through enzymatic Ang‐I processing and Ang‐II‐mediated internalization.^[^ ^9^ ^]^

We designed particles that carried EXP3174, an angiotensin‐II type 1 receptor (AT1R) ligand, in the NP corona to mediate receptor attachment (**Figure** **1**). As a G protein‐coupled receptor (GPCR) antagonist, it has the advantage that binding cannot trigger cellular NP uptake,^[^ ^25^ ^]^ but only membrane binding^[^ ^26^ ^]^ and thus prevents particle uptake by off‐target cells that only carry the AT1R. For the second recognition criterion we outfitted the particles with the ability to probe cell surfaces for the presence of angiotensin converting enzyme (ACE) which recognizes the proligand angiotensin‐I (Ang‐I) in the particle corona and converts it to the active ligand angiotensin‐II (Ang‐II). As a third recognition step, Ang‐II binds to the AT1R and, as an agonist, triggers cell uptake of particles upon receptor binding.^[^ ^25^ ^]^ The whole process of target cell recognition can best be illustrated with a flow chart (Figure S1, Supporting Information).

We examined the particles for their target receptor avidity and target‐cell specificity in vitro. Additionally, we assessed how the simultaneous presentation of two ligands addressing the same receptor, an antagonist promoting cell membrane binding, and an agonist, supporting cellular internalization, would affect the NPs ability to mediate cellular uptake. Lastly, we investigated if particles with such a virus‐mimetic triple recognition strategy were superior to conventional NPs or particles mediating only either the viral attachment or stepwise internalization for reaching MCs in vivo.

## 2 Results

### 2.1 Block‐Copolymers Allow for a Virus‐Mimetic Particle Design

**Figure 2:**
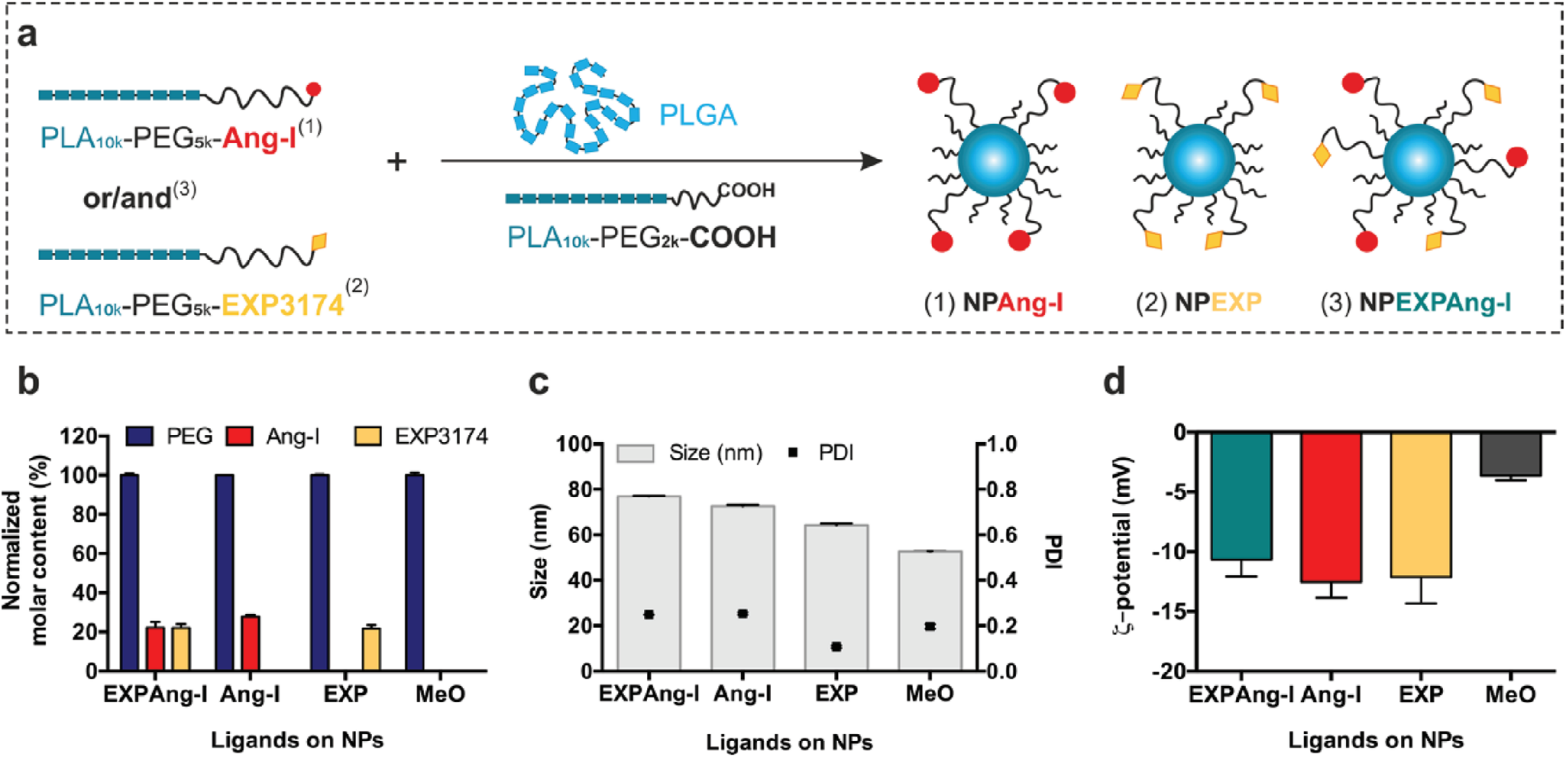
NP characterization. a) Assembly of ligand‐decorated NPs. b) Molar ligand content of different NP species normalized to the PEG content, c) size and polydispersity index (PDI), and d) *ς*‐potential of the resulting NP formulations. Results are presented as mean ± SD of at least *n* = 3 measurements.

For the development of virus‐mimetic NPs we coupled the ligands EXP3174 and Ang‐I, to poly(ethylene glycol)_5k_‐poly(lactic acid)_10k_ (PEG‐PLA) block copolymers (Figure S2, Supporting Information), which were blended with poly(lactic‐co‐glycolic acid) (PLGA) for NP manufacturing via bulk nanoprecipitation^[^ ^27^ ^]^ rendering particles with sufficient stability in vivo.^[^ ^28^ ^]^ Such NPs are known for their excellent biocompatibility and highly tunable composition (**Figure** **2**). The remaining, non‐functionalized polymers were carboxylic acid‐ended PEG‐PLA with a shorter 2k PEG and a 10k PLA block (COOH‐PEG_2k_‐PLA_10k_) (Figure 2a). By modifying the polymers with ligands prior to NP preparation, the ligand density can be precisely controlled. Particles were prepared such that 20% of their PEG chains were decorated with Ang‐I and an additional 20% with EXP3174 (NPEXPAng‐I) (Figure 2b). The ligand density was kept at a 40% maximum to avoid stearic hindrance among ligands and non‐specific interactions.^[^ ^29^ ^]^ As a control, ligand‐free methoxy‐PEG‐terminated particles (NPMeO) and particles carrying either 20% Ang‐I or EXP (NPAng‐I and NPEXP, respectively) were assembled (Figure 2a). By combining long ligand‐carrying polymers with shorter non‐functionalized polymers for particle preparation, the size of the NPs could be kept under 80 nm (Figure 2c) to endow particles with the ability of passing through the endothelial fenestrations of mesangial capillaries.^[^ ^30^ ^]^ Carboxylic acid terminated block copolymers were selected as a filler that provides an overall negative particle charge ideal to avoid non‐specific electrostatic adsorption to the negative cell membranes^[^ ^31^ ^]^ (Figure 2d).

### 2.2 NPs Recognize Target Receptors In Vitro

**Figure 3:**
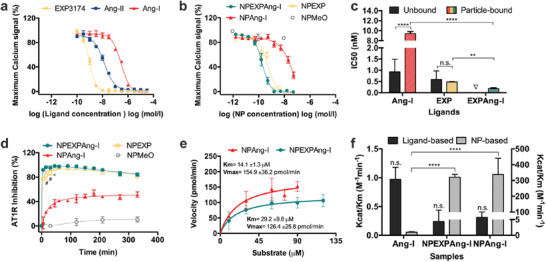
In vitro interaction with AT1R and ACE. Interaction of ligand‐decorated NPs with their target AT1R (a–d) and ACE (e,f) determined by intracellular calcium measurements. a) Ligand affinity and b) particle avidity for the AT1R. c) IC50 values for the free and particle‐bound ligands. d) Kinetic measurement of the AT1R inhibition by ligand‐decorated particles. e) Michaelis–Menten kinetics of NPEXPAng‐I and NAng‐I. f) Specificity constant (*k*_cat_/*K*_M_) for the free and particle‐bound Ang‐I calculated based on ligand and NP concentration. Results are presented as mean ± SD of at least *n* = 3 measurements. Levels of statistical significance are indicated as **p ≤ 0.01, ****p ≤ 0.0001, and #*p* ≤ 0.0001 and *x* ≤ 0.001 comparing the AT1R inhibition of NPEXP and NPEXPAng‐I at different time points. n.s.: non‐significant. ∇: a mixture of free Ang‐I and EXP3174 was not investigated since both ligands compete for the same receptor.

Particle avidity for the target receptor, which mediates primary attachment and subsequent internalization, was investigated using calcium mobilization assays (**Figure** **3**), since the stimulation or silencing of the Gq‐coupled AT1R with an agonist or antagonist results in a cytosolic Ca^2+^ influx or its suppression, respectively.^[^ ^32^ ^]^ To that end, target‐positive rat MCs (rMCs)^[^ ^9^ ^]^ were incubated with varying concentrations of either free ligands or NP‐formulations prior to stimulation with Ang‐II and recording the resulting calcium signal. As depicted in Figure 3a, control experiments with free EXP3174 and Ang‐II revealed a high affinity of both compounds for the AT1R in the low nanomolar range (IC50 values of 0.6 ± 0.4 and 1.5 ± 0.1 nm, respectively) which is in accordance with literature values.^[^ ^33^ ^]^ Ang‐I displays a lower affinity (IC50 0.9 ± 0.6 *μ* m), as the receptor binding and activation occurs only after enzymatic conversion to Ang‐II by ACE present on the cell membrane. The coupling of ligands to linkers leads to an affinity loss that is compensated by the high avidity multivalent binding of several receptors simultaneously^[^ ^26^ ^]^ (Figure 3b,c). NPAng‐I show a lower avidity for the AT1R (IC50 of 9.4 ± 0.4 nm) than NPEXP (IC50 of 0.4 ± 0.1 nm), as their primary interaction is with the ACE. Nevertheless, particle binding of Ang‐I leads to a significant decrease in IC50 values compared to the free ligand due to a facilitated enzymatic cleavage at the NP‐interface^[^ ^34^ ^]^ and the subsequent multivalent binding. NPEXP in contrast, had avidities that were of the same order of magnitude as for the free ligand. Interestingly, particles that carried both ligands, NPEXPAng‐I, showed a cooperative effect with respect to receptor interaction, as they had significantly higher avidity for the AT1R (IC50 of 0.2 ± 0.09 nm) than either of the particles carrying only one type of ligand (Figure 3c). This proves that the ligands do not hinder each other's interaction, which was a point of great concern, as after Ang‐I enzymatic activation to Ang‐II both ligands target the same receptor in a simultaneous agonistic and antagonistic manner. NPMeO confirmed that the assay was ligand‐specific, as they did not elicit any response due to their lack of functionalization (Figure 3b).

To assess kinetics of cell/particle interactions, intracellular calcium measurements were performed over a 5.5‐h period. The extent to which they could silence calcium signaling triggered by the present free agonist served as a measure for the completeness to which the respective particles had bound via their ligands to the AT1R in the cell surface at different time points (Figure 3d). Particles carrying only Ang‐I on their surface displayed a slow receptor binding since they initially need to be activated by the cell membrane‐bound ACE to Ang‐II carrying particles before they can interact with the AT1R. The receptor binding reached a maximum at about 40% after 1‐h incubation, which remained constant over the assay's duration. This points towards a fast internalization of the particles once a certain number of proligand is activated. Once Ang‐II on the particle surface binds to a receptor, the particles are rapidly internalized (as they have picomolar AT1R avidities^[^ ^9^ ^]^) which indicates that not all proligands may need to be activated for NP internalization to occur. This phenomenon is avoided when adding EXP3174 as an attachment factor on the particle surface. A very fast and complete receptor blockage occurs after only 5 min of particle incubation (for NPEXPAng‐I and NPEXP alike). The AT1R inhibition is maintained over almost the whole measurement and descends to about 80% at the last time points, probably due to receptor upregulation and recycling.^[^ ^35^ ^]^ The attachment by EXP3174 to the cell membrane slows down the recognition process and enables a higher Ang‐I to Ang‐II activation that can more efficiently bind to the AT1R. Comparing NPEXPAng‐I and NPEXP there is a significantly higher initial AT1R inhibition of NPEXPAng‐I which evens out after 45 min. This is probably due to the combined effect of the two ligands which leads to a higher avidity for the AT1R, as seen previously (Figure 3c).

A prerequisite for particle internalization is the ability of ACE to activate Ang‐I to Ang‐II. Therefore, we investigated the enzyme kinetics for NPEXPAng‐I, to determine whether the presence of the antagonist on the particle surface would hinder the enzymatic reaction. A soluble form of ACE was incubated for varying time periods with different particle concentrations and the resulting Ang‐II on the NP corona was quantified running calcium mobilization assays. The interference of the EXP3174 ligand in the assay was assessed by measuring the signal inhibition exhibited by NPEXP (Figure S3, Supporting Information). The Michaelis–Menten constant (*K*_M_) determined for both NPAng‐I and NPEXPAng‐I (Figure 3e), resulted in values that were of the same order of magnitude as for the free ligand^[^ ^9^, ^36^ ^]^ for both particle formulations. Additionally, we determined the catalysis constant (*k*_cat_) to calculate the specificity constant (*k*_cat_/*K*_M_) which is a useful indicator for comparing the affinity of different substrates for the same enzyme^[^ ^37^ ^]^ (Figure 3f). The enzymatic activation of Ang‐I on the NPEXPAng‐I corona was not significantly different from the one on NPAng‐I, indicating that ACE is not sterically hindered by the additional ligand EXP3174. Furthermore, the *k*_cat_/*K*_M_ value calculated based on the ligand concentration was equal for free and particle‐bound Ang‐I. More so, when *k*_cat_/*K*_M_ is calculated based on the NP concentration the bound ligand is a significantly better substrate for the enzyme, which is a result of the binding of several ligand molecules on the particle surface to various enzyme molecules (Figure 3f).

### 2.3 Virus‐Mimetic NPs Are Target‐Cell Specific

**Figure 4:**
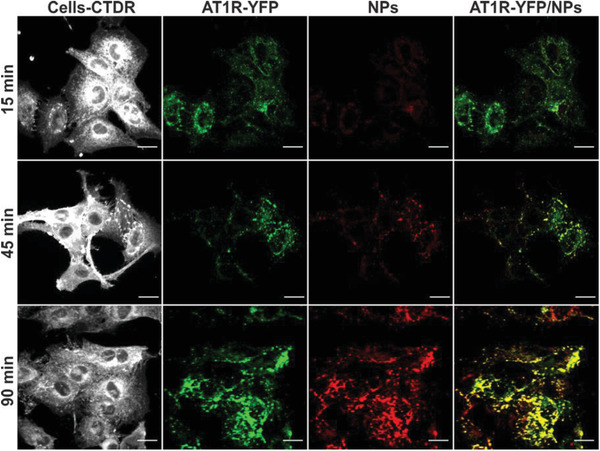
Cellular localization of NPEXPAng‐I. NPEXPAng‐I (red) are internalized in target cells (white) transfected with a YFP‐tagged AT1R (green) (pAT1R‐rMCs) at different incubation times. Scale bar: 20 µm.

After the particle interaction with their individual targets had been successfully established, the next step was to determine if NPs carrying an antagonist as well as an agonist on their corona would still trigger internalization by their target cells, and if so, if the uptake ensued from a specific ligand‐receptor interaction. As antagonists do not cause AT1R‐mediated endocytosis^[^ ^26^, ^38^ ^]^ and agonists do,^[^ ^9^, ^39^ ^]^ we investigated via confocal laser scanning microscopy (CLSM) the cellular localization of NPEXPAng‐I in rMCs expressing YFP‐tagged AT1R (pAT1R‐rMCs). As shown in **Figure** **4**, NPEXPAng‐I‐associated fluorescence was found inside the cells. It increased with higher incubation times and strongly colocalized with the AT1R fluorescence. This points towards a specific particle uptake, mediated by the AT1R. However, particles carrying only the antagonist (NPEXP) were not internalized by the cells and located mostly on the cellular surface (Figure S4A, Supporting Information), which is in accordance with previous findings with EXP3174 and other antagonist‐decorated particles.^[^ ^26^, ^38^ ^]^ But still, the particle fluorescence also colocalized with the receptor fluorescence, demonstrating a receptor‐mediated attachment. As NPAng‐I were also internalized by the cells (Figure S4B, Supporting Information), the enzymatically created Ang‐II seems to mediate the cellular uptake of NPEXPAng‐I. Interestingly, there was a rearrangement of the receptors on the cellular membrane with increasing incubation times (Figure 4), from a more diffuse and uniform cell membrane distribution (15 min) to a more concentrated clustering (90 min), which strongly colocalized with the NP fluorescence. This is additional evidence that the uptake is mediated by the AT1R, as the activation of receptors that are internalized via clathrin‐coated pits, such as the GPCR like the AT1R, promotes receptor clustering.^[^ ^40^, ^41^ ^]^ For NPEXP a receptor rearrangement on the cell membrane also occurred, which is a result of a multivalent receptor binding promoted by receptor movement on the cellular surface. Once NPEXP attach to a receptor on the cell membrane, their lack of internalization can lead to receptor‐particle mobility on the cell membrane, and further receptor binding.^[^ ^42^ ^]^ NPMeO were not taken up by the cells (Figure S4c, Supporting Information), confirming that a specific targeting mechanism is essential to mediate a high cellular internalization.

**Figure 5:**
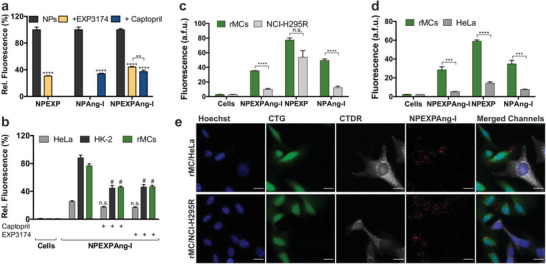
Uptake specificity of virus‐mimetic NPEXPAng‐I. a) Ligand‐mediated internalization of NPEXPAng‐I, NPAng‐I, and NPEXP in rMCs inhibited by free EXP3174 and captopril. b) Uptake of NPEXPAng‐I in AT1R and ACE positive rMCs and HK‐2 cells and AT1R and ACE negative HeLa cells. Specificity of particle uptake in co‐culture of target rMCs with off‐target c) NCI‐H295‐R cells or d) HeLa cells analyzed via flow cytometry. e) CLMS images of particle uptake (red) in green‐stained (CTG) rMCs (green) in co‐culture with deep red‐stained (CTDR) off‐target HeLa or NCI‐H295R cells (white). Scale bar: 20 µm. Results are presented as mean ± SD of at least *n* = 3 measurements. Levels of statistical significance are indicated as **p ≤ 0.01, ***p ≤ 0.001, ****p ≤ 0.0001, and as #*p* ≤ 0.0001 comparing the uptake of NPs in cells with and without captopril or EXP3174 inhibition. n.s.: non‐significant.

Overall, we could demonstrate that the presence of an attachment‐mediating antagonistic ligand linked to the particle corona does not hinder subsequent particle internalization. More so, the inclusion of an additional ligand on the particle surface compensated the targeting loss due to steric hindrance of the Ang‐I ligand by the addition of a higher number of long polymer chains (Figure S5, Supporting Information). To further confirm the particle specificity and ligand‐mediated internalization, the cells were pre‐incubated for 30 min prior to particle addition with free EXP3174 or captopril, an ACE inhibitor, which resulted in a suppression of the particle‐associated fluorescence analyzed by flow cytometry (**Figure** **5**a) and CLSM (Figure S6, Supporting Information).

As the simultaneous presentation of two ligands on a particle surface can lead to more off‐target interactions, we examined the particle internalization in different cell lines by flow cytometry (Figure 5b). HeLa cells, which do not express ACE and only express minor AT1R levels^[^ ^9^, ^38^ ^]^ showed a low particle uptake, which was non‐specific as it could not be suppressed by captopril or EXP3174. On the contrary, rMCs and HK‐2 cells expressing both the targets^[^ ^9^ ^]^ were able to take up the particles, shown by the much higher particle‐associated cell fluorescence. The internalization was also mediated by the activated proligand binding to the AT1R, as the preincubation of cells with captopril or EXP3174 significantly suppressed the cell fluorescence. This is a great indicator of particle specificity for their target cells. Nevertheless, when NPs enter the body, they are presented simultaneously with target and off‐target cells. Therefore, we investigated if our NPEXPAng‐I were able to differentiate between them when presented simultaneously. Target cells (rMCs) were seeded together with an excess of off‐target NCI‐H295R or HeLa cells, which both lack the ACE and express high and low AT1R levels, respectively.^[^ ^9^, ^38^ ^]^ They were incubated with the different NP formulations and investigated for particle‐associated fluorescence through flow cytometry (Figure 5c,d). NPEXPAng‐I showed outstanding target cell specificity, as they accumulated significantly more in target rMCs. The specificity is conferred by Ang‐I as NPAng‐I showed also low accumulation in both off‐target cells. On the contrary, NPEXP bound to the cell surface to the same degree in rMCs as in NCI‐H295R cells, which express high AT1R levels, demonstrating that a simple one‐step recognition process is not enough to confer particle selectivity. CLSM images confirmed our flow cytometry findings (Figures 5e; Figure S7, Supporting Information), were NPEXPAng‐I‐ and NPAng‐I‐fluorescence (red) was mostly associated with target rMCs (green) and not in off‐target HeLa or NCI‐H295R cells (white), while NPEXP fluorescence was found in both rMCs and AT1R‐expressing NCI‐H295R cells. Taken all together, these results demonstrate that the NPEXPAng‐I uptake is receptor‐mediated and that the initial cell attachment through the EXP3174 ligand does not reduce the particle specificity for the target cells conferred by the virus‐mimetic recognition process.

### 2.4 Virus‐Mimetic NPs Target MCs In Vivo

**Figure 6:**
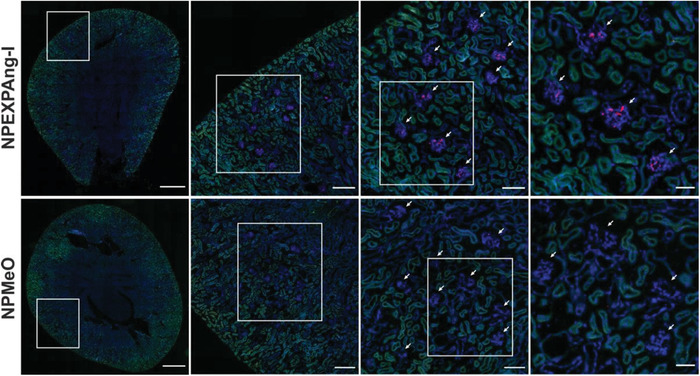
Distribution of NPEXPAng‐I and NPMeO in mice kidney. Glomeruli are indicated with white arrows. Blue: DAPI staining of cell nuclei; green: tissue autofluorescence; red: NP‐associated fluorescence. From left to right squared out regions are shown as magnifications. Scale bars left to right: 900, 200, 100, and 50 µm.

Since the complementary targeting ability of both ligands on NPEXPAng‐I and the particle specificity was demonstrated in vitro the next step was to determine whether the viral recognition principle would lead to a higher in vivo MC accumulation. To that end, targeted (NPEXPAng‐I, NPAng‐I, and NPEXP) (Figure 1a) and non‐targeted (NPMeO) particle formulations were injected into NRMI mice and cryosections of the kidneys examined for particle‐associated fluorescence (**Figure** **6**; Figure S8a, Supporting Information). As depicted in Figure 6, NPEXPAng‐I fluorescence could be found homogeneously over all glomeruli in the kidney section, with no fluorescence in other kidney structures, such as the tubuli. On the contrary, for NPMeO almost no NP fluorescence could be detected in the kidney sections. This demonstrates that simple size‐dependent targeting is not enough to achieve a particle accumulation in MCs and NPMeO are probably cleared out of the mesangium due to their lack of specific cellular interaction. More so, NPEXP which are targeted NPs but not able to mediate cellular internalization also depicted very little glomerular fluorescence (Figure S8a, Supporting Information), demonstrating that particle uptake is fundamental to achieve a high MC accumulation. Furthermore, NPEXPAng‐I showed a much stronger and homogeneous glomerular distribution than NPAng‐I, which lack the attachment factor (Figure S8a, Supporting Information), demonstrating that in vivo an enhanced target cell recognition principle is highly advantageous.

**Figure 7:**
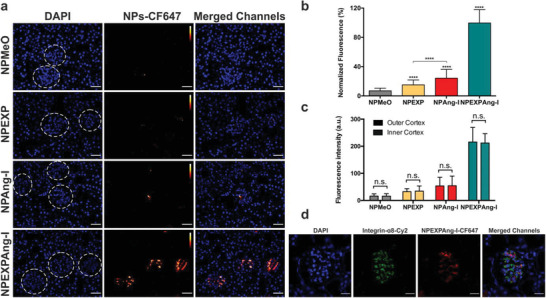
Assessment of the NP‐associated fluorescence detected in kidney glomeruli analyzed through fluorescence microscopy. a) Images of kidney glomeruli (dotted circles) of mice treated with the different particle formulations. Scale bar: 40 µm. b) Quantitative analysis of the complete glomerular NP‐fluorescence. c) Comparison of the particle‐associated fluorescence in the glomeruli of the outer and inner cortex. d) Glomerular localization of NPEXPAng‐I determined via CLSM and Integrin‐*α*8‐staining of MCs. Scale bar: 20 µm. Results in (c) and (d) are presented as mean ± SD of at least *n* = 120 glomeruli fluorescent measurements of *n* = 6 mice per NP sample. Levels of statistical significance are indicated as ****p ≤ 0.0001. n.s.: non‐significant.

In order to quantitatively assess the NP‐associated fluorescence and better distinguish the differences among the different particle formulations, images of the glomeruli were taken at higher magnifications (**Figure** **7**a). Quantitative analysis of the glomerular fluorescence yielded a 15‐fold higher fluorescence for virus‐mimetic particles with enhanced recognition mechanism (NPEXPAng‐I) compared to non‐targeted control particles (NPMeO), which showed only small fluorescence spots in some glomeruli. Additionally, NPEXPAng‐I displayed significantly higher accumulation than one‐ligand targeted particles (7‐ and 5‐fold higher than NPEXP and NPAng‐I, respectively) (Figure 7b). That the detected florescence was particle‐associated, was confirmed by the kidney distribution of the free dye used for particle labelling (CF647), which showed strong tubular but no glomerular fluorescence (Figure S8b, Supporting Information), as due to its small size it can be freely filtrated. To assess the NP glomerular distribution the fluorescence of the glomeruli in the outer and inner cortex was compared (Figure 7c). For all particle formulations there were no significant differences among the two populations. This indicates that the particles are distributed homogeneously in the glomeruli of the entire kidney cortex, which is an indispensable prerequisite for the treatment of glomerular‐associated diseases. Finally, as besides MCs there are other cells in the glomerulus which could have internalized the NPs, a specific antibody‐staining of MCs using integrin‐*α*8 as a marker was performed to ascertain that the particles accumulated in MCs. As depicted in Figure 7d, the NPEXPAng‐I fluorescence localized inside the antibody‐stained MCs, confirming that our particles were able, not only to reach the glomerular mesangium, but also to be taken up by MCs.

Taken together these results clearly show that size‐mediated targeting is a necessary prerequisite to reach the mesangium, but insufficient to achieve particle accumulation in MCs. NP internalization seems to be imperative to avoid mesangial clearance, which explains that particles lacking this trait (NPMeO and NPEXP) lead to the lowest glomerular fluorescence. Implementing a virus‐mimetic recognition principle (NPAng‐I) increases NP specificity and results in particle uptake which in turn leads to a higher MC‐accumulation. However, facilitating the target cell recognition via an initial virus‐like cell attachment (NPEXPAng‐I) significantly enhances the NP's targeting potential, a result of a combined effect of the two ligands, as shown by the in vitro studies. Furthermore, the enhanced functionalization of NPEXPAng‐I does not lead to a decrease in the particle blood residence. Generally, NPs are coated with polymers such as PEG, which increase their circulation time^[^ ^43^ ^]^ and decrease plasma protein adsorption.^[^ ^44^ ^]^ A positive effect, which is usually counteracted by ligand functionalization, as off‐target cells expressing the targeted receptors can bind and interfere with the NPs. Nevertheless, quantification of the plasma NP fluorescence one hour after injection showed that NPEXPAng‐I remained in circulation to the same extent as non‐targeted NPMeO and significantly longer than the other targeted formulations (Figure S8c, Supporting Information). This is probably due to a higher particle specificity resulting from a more complex cell recognition process. Overall, our results demonstrate that by closely mimicking the viral attachment and internalization and combining it with an optimal NP size we were able to develop NPs that target and accumulate in MCs.

## 3 Discussion

The presented results are of immediate relevance for the delivery of drugs to MCs. Even though, previous studies found that nanomaterials enter the mesangium, they had been carried out mainly under pathological conditions,^[^ ^45^, ^46^, ^47^, ^48^, ^49^, ^50^ ^]^ associated with an increased vascular permeability and inflammation.^[^ ^51^ ^]^ This explains why the NPs that were used had diameters as big as 400 nm^[^ ^52^ ^]^ which is far above the 80 nm size limit of endothelial fenestration permeability found under physiological conditions.^[^ ^22^ ^]^ Even though such particles might be beneficial to deliver drugs to halt tissue damage once a certain disease state is established, they may not be useful to prevent its outbreak. In the initial phases of DN, for example, there are little morphological changes which can hinder mesangial particle deposition.^[^ ^53^ ^]^ In contrast, the particles we suggest, would enable a high NP accumulation under physiological conditions which could be used to stop or slow down disease development in an early phase. As follow up experiments, it would be interesting to investigate how the particles behave in a diabetic animal model, where the target expression levels could vary. In this regard, a higher particle MC‐accumulation would be expected due to an overexpression of ACE under diabetic conditions.^[^ ^54^ ^]^ Another field of application could be the control of vascular endothelial growth factor type A regulation (VEGF‐A) inside glomeruli. It has been shown that unphysiologically high or low VEGF‐A levels can cause renal disease.^[^ ^55^ ^]^ MCs could be used to balance these levels by recombinant VEGF‐A production or antiangiogenic drugs.

Beyond these rather concrete therapeutic implications our findings also shed light on the need for a more rigorous NP design for cell identification in vitro and in vivo. NP biodistribution following systemic administration always entails a high material loss to clearance organs, such as the liver and the spleen^[^ ^56^ ^]^ which is also the fate of virus particles.^[^ ^57^ ^]^ Since NPs are distributed in the organism typically by passive transport mechanisms, their appearance in a specific tissue is a matter of their physicochemical properties. However, the fraction of particles accumulating in a tissue can be increased if they are able to actively interact with the cells of interest. Thereby, it is not sufficient to outfit NPs with one or more ligands that bind to respective receptors to confirm a cell's identity. Our particles demonstrate quite clearly that viral strategies of a stepwise cell identification are more advantageous. More so, it is not enough to only mimic the sequential viral internalization (NPAng‐I). Our results show that incorporating an additional attachment step (NPEXPAng‐I) is necessary to increase the target tissue in vivo accumulation. This confirms that the viral attachment is not only essential for viral cell recognition but also for optimal nanomaterial targeting. Furthermore, performing the attachment through an antagonist for the same receptor that is responsible in a subsequent step for agonist‐mediated particle uptake does not impede NP internalization.

For cell identification, receptors that belong to the family of GPCRs, such as the AT1R we used in this study, are of particularly high value since the consequences of a positive outcome of an individual cell check can be determined by choosing the type of ligand that is used for the explorative interaction.^[^ ^25^ ^]^ Thus, by carefully selecting the targets we use for the interaction and the type of ligand, we can outfit particles with a logic that may allow for an identification of even more concealed target cells than we investigated in this study. An example could be local ocular applications in retinal tissue in which a particle would need to be able to distinguish between the more than 60 cell types that are present.^[^ ^58^ ^]^

## 4 Conclusion

We could show that virus‐mimetic NPs that triple check cell identity are subject to an enhanced NP accumulation in the targeted MCs in vivo. By combining an antagonistic ligand, mimicking initial cell attachment of viruses, with an enzyme‐mediated target cell recognition process, our particles had an outstandingly high in vitro target avidity together with an exceptional target cell specificity. We could also demonstrate that the simultaneous hetero‐multivalent binding of a particle‐tethered agonist and antagonist for the same GPCR leads to particle uptake which, to the best of our knowledge, has never been shown before. Overall, our results suggest that non‐specific size‐mediated passive targeting is not sufficient to achieve a satisfactory particle accumulation in MCs. Even traditional particle functionalization with a single ligand appears to be an insufficient approach. However, by mimicking the intricate multistep viral target cell binding and recognition process we obtained particles that are able to identify and accumulate in MCs. This will open new options for the delivery of drugs for the treatment of renal diseases, such as DN for which we are lacking an efficient therapy.

## 5 Experimental Section

##### Materials

Carboxylic‐acid and Boc‐amine‐ or methoxy‐functionalized PEG (2000 and 5000 Da) were purchased from JenKem Technology USA Inc. (Allen, TX, USA). Lysine N‐modified Ang‐I and Ang‐II (Lys‐Ang‐I and Lys‐Ang‐II) (purity >98%) were purchased from Genscript (Piscataway, NJ, USA). EXP3174 and captopril were obtained from Santa Cruz Biotechnology (Dallas, TX, USA). Fura‐2AM was purchased from Life Technologies (Carlsbad, CA, USA). DPBS, Lipofectamine 2000, TAMRA‐amine, Pierce BCA Assay kit, CellMask Deep Red Plasma Membrane Stain (CMDR), CellTracker Deep Red (CTDR), and Green (CTG) were purchased from Thermo Fisher Scientific (Waltham, MA, USA). Hoechst 33258 was obtained from Polysciences Inc. (Warrington, PA, USA). Integrin‐*α*8 goat antibody was purchased from R&D Systems (Minneapolis, MN, USA). Cy2‐anti‐goat secondary antibody was obtained from Jackson Immuno Research Labs (PA, USA). CXN2‐HA‐AT1R‐YFP was a gift from Yusuke Ohba (Addgene plasmid #101659; http://n2t.net/addgene:101659; RRID: Addgene_101659). All other materials and reagents in analytical grade were obtained from Sigma‐Aldrich (Taufkirchen, Germany).

##### Cell Culture

rMCs were kindly gifted by Prof. Armin Kurtz (Institute of Physiology, University of Regensburg). NCI‐H295R (CRL‐2128) and HeLa (CCL‐2) cells were purchased from ATCC. All three cell lines were cultured in RPMI1640 medium supplemented with 10% fetal bovine serum (FBS) (Biowest, Nuaillé, France), insulin‐transferrin‐selenium, and 100 nm hydrocortisone. HK‐2 cells were purchased from ATCC (CRL‐2190) and maintained in DMEM‐F12 (1:1) medium supplemented with 10% FBS. pAT1R‐rMCs were obtained by transfecting rMCs with a plasmid encoding the AT1R with a YFP‐tag (CXN2‐HA‐AT1R‐YFP) using Lipofectamine 2000 after the manufacturer´s instructions. pAT1R‐rMCs were cultured in RPMI1640 medium supplemented with geneticin (600 µg mL^−1^).

##### Polymer Preparation

PEG‐PLA block‐copolymers were synthesized after Qian et. al^[^ ^59^ ^]^ with slight modifications as previously described.^[^ ^9^, ^60^ ^]^ For the preparation of Ang‐I‐modified polymers, COOH‐PEG_5k_‐PLA_10k_ (14 µmol) was activated with 1‐ethyl‐3‐(3‐dimethylaminopropyl)carbodiimide and N‐hydroxysuccinimide (NHS) (350 µmol) in *N*,*N*‐dimethylformamide (DMF) for 2 h. Afterward, 2‐mercapthoethanol (863 µmol) was added (20 min), prior to the dropwise addition of *N*,*N*‐diisopropylethylamine (DIPEA) (66 µmol) and Lys‐Ang‐I (17 µmol). After 48 h, the resulting polymer was diluted in ultrapure water (DMF concentration below 10%) and dialyzed using a 6–8 kDa molecular weight cut‐off regenerated cellulose dialysis membrane over 24 h. For EXP3174‐modified polymer preparation, EXP3174 (96.4 µmol) was activated with *N*,*N*'‐dicyclohexylcarbodiimide and NHS (96.4 µmol) in DMF for 2 h. Afterward resulting urea byproducts were removed by centrifugation (5 min, 12 000 × *g*) and filtration with a 0.2 µm Rotilabo PTFE syringe filter. NH_2_‐PEG_5k_‐PLA_10k_ (27.6 µmol) in DMF and DIPEA (1.7 mmol) were added to the activated ligand and reacted over 20 h. The ligand‐modified polymer was purified by precipitation in ice cold 1:5 (v/v) diethyl‐ether:methanol and dialysis against 10% ethanol in 10 mm borate buffer (pH 8.5) over 24 h followed by dialysis against ultrapure water over 24 h using a 6–8 kDa molecular weight cut‐off regenerated cellulose dialysis membrane. Ligand‐modified block‐copolymers were lyophilized over 72 h prior to ligand‐coupling confirmation (Figure S2, Supporting Information). Polymer characterization was performed through ^1^H‐NMR using a Bruker Avance II 400 spectrometer (Bruker BioSpin GmbH, Rheinstetten, Germany) (Figures S9–S14, Supporting Information) and high‐performance liquid chromatography (HPLC), using an Agilent Infinity 1260 HPLC (Agilent Technologies GmbH, Waldbronn, Germany) (Figure S15, Supporting Information).^[^ ^61^ ^]^

For particle detection, TAMRA‐amine (for CLSM) and CF6467‐amine (for flow cytometry and in vivo experiments) were covalently coupled to carboxylic acid‐terminated 13.4 kDa PLGA prior to particle preparation as previously described.^[^ ^62^ ^]^

##### NP Preparation and Characterization

PEG‐PLA block‐copolymers and 13.4 kDa PLGA were mixed at a 70:30 mass ratio to a final concentration of 10 mg mL^−1^ in ACN. For ligand‐modified particles COOH‐PEG_2k_‐PLA_10k_ and ligand‐modified polymers were mixed accordingly so that 20% of the polymers making up the NP‐structure were modified with Ang‐I (NPAng‐I) or/and EXP3174 (NPEXP and NPEXPAng‐I, respectively). NPs were prepared via bulk nanoprecipitation of polymer mixtures in vigorously stirring 10% DPBS (v/v) (pH 7.4) to a final concentration of 1 mg mL^−1^. Particles were stirred for 2 h and concentrated by ultracentrifugation using a 30‐kDa molecular weight cutoff Microsep advance centrifugal device (Pall Life Sciences) for 20 min at 756 × *g*.

Size and *ξ*‐potential of the resulting particles were determined in 10% phosphate buffered saline (PBS) (v/v) at a constant temperature of 25 °C using 1 or 3.5 mg mL^−1^ concentrations, respectively, with a ZetaSizer Nano ZS (Malvern Instruments).^[^ ^9^, ^60^ ^]^ Quantification of particle PEG concentration was performed using a colorimetric iodine complexing assay^[^ ^63^ ^]^ and correlated with the gravimetrical NP content determined via lyophilization as previously described.^[^ ^62^ ^]^ The molar particle concentration was calculated from the particle mass determined through the colorimetric iodine complexing assay, the particle density (1.25 g cm^−3^)^[^ ^64^ ^]^ and the hydrodynamic diameter of the NPs obtained through DLS measurements assuming a spherical particle shape. Ang‐I concentration on the particle corona was quantified using a Pierce BCA assay kit, after the manufacturer's instructions, and a FLUOstar Omega microplate reader (BMG Labtech). EXP3174 concentration was determined fluorometrically (*λ*_ex_ = 250 nm and *λ*_em_ = 370 nm) using a LS‐5S fluorescence plate reader (PerkinElmer).

##### Intracellular Calcium Measurements

The AT1R interaction of the different NP formulations was assessed through a ratiometric Fura‐2 Ca^2+^ chelator method^[^ ^65^ ^]^ as previously described^[^ ^9^, ^26^ ^]^ using AT1R positive rMCs.^[^ ^9^ ^]^ To determine the particle avidity and ligand affinity for the AT1R, Fura‐2‐loaded‐rMC in suspension (45 µL ≈ 90 000 cells) were incubated with different samples (10 µL) (NPs or free ligands ranging from 1–300 *μ* m ligand based concentration) for 30 min. Afterward, cells were stimulated with Lys‐Ang‐II (45 µL = 300 nm) and the resulting calcium signal immediately recorded for 1 min using a FLUOstar Omega microplate reader with 340/20 nm and 380/20 nm excitation and 510/20 nm emission bandpass filters. To determine the kinetics of the AT1R interaction the same procedure was used, but the samples (10 *μ* m ligand concentration) were incubated for different time periods (5–320 min) with the cells.

##### Enzyme Kinetic Measurements

The Michaelis–Menten kinetics were determined as previously described.^[^ ^9^ ^]^ In order to rule out the interference of the EXP3174 ligand on NPEXPAng‐I under the experimental conditions used, NPEXP was used as a control (Figure S3, Supporting Information). The *K*_M_ for particle‐ and ligand‐based concentrations, *k*_cat_ and *k*_cat_/*K*_M_ were obtained using GraphPad Prism 6.0.

##### Cellular Distribution of NPs

In order to determine the cellular distribution of the different particle formulations (Figure 4), pAT1R‐rMCs were seeded into 8‐well *μ*‐slides (Ibidi, Graefelfing, Germany) (10 000 cells per well) and incubated over 24 h. Then they were incubated with pre‐warmed NP‐solutions (0.2 mg mL^−1^) in Leibovitz medium (LM) supplemented with 0.1% bovine serum albumin (BSA) for 15, 45, or 90 min. Afterward, the NPs were discarded, and the cells washed thoroughly with DPBS prior to cell staining with 1× CMDR for 5 min and fixation with 4% paraformaldehyde (PFA) in DPBS for 10 min. Confocal images were acquired using a Zeiss LSM 700 microscope with the focal plane set at 1.4 µm using the Zen Software (Carl Zeiss Microscopy). For particle uptake and binding inhibition cells were preincubated with free EXP3174 (1 mm) prior to particle addition. Images were analyzed using Fiji software.^[^ ^66^ ^]^ Particle uptake through flow cytometry was performed as previously described.^[^ ^9^ ^]^ In short, rMCs were seeded in 24‐well plates at a density of 30 000 cells per well and incubated for 48 h (37 °C). Prewarmed NP‐solutions (0.7 mg mL^−1^ in LM with 0.1% BSA) were added to the cells, after washing them with DPBS, and incubated for 45 min. To confirm the uptake specificity, cells were incubated with 1 mm of captopril and/or EXP3174 for 30 min prior to particle addition. Afterward, particle solutions were discarded, and the cells washed thoroughly with DPBS, trypsinized and centrifuged (2×, 200 × *g* 5 min, 4 °C). NP‐associated cell fluorescence was analyzed in DPBS using a FACS Calibur cytometer (Becton Dickinson). Fluorescence was excited at 633 nm and recorded using a 661/16 nm bandpass filter. The population of viable cells was gated using Flowing software 2.5.1. (Turku Centre for Biotechnology) and the geometric mean of the NP‐associated fluorescence was analyzed.

##### NP Target Cell Specificity

To assess the NP uptake in different cells lines through flow cytometry rMCs, HK‐2 and HeLa cells were seeded out in 24‐well plates at a density of 30 000, 50 000, or 100 000 cells per well, respectively and incubated over 48 h. Afterwards pre‐warmed NP‐solutions (0.7 mg mL^−1^ in LM with 0.1% BSA) were added on top of the cells and processed as described above. The particle specificity in co‐culture of target and off‐target cells was investigated through CLSM and flow cytometry analysis as previously described.^[^ ^9^ ^]^

##### NP Kidney Distribution In Vivo

The experimental procedures on animals were carried out according to the national and institutional guidelines and were approved by the local authority (Regierung von Unterfranken, reference number: 55.2‐2532‐2‐329). As model animals 10‐week‐old female NMRI‐mice (Charles River Laboratories, Sulzfeld, Germany) were used. The different NP formulations (NPEXPAng‐I, NPAng‐I, NPEXP, and NPMeO) (120 nm NPs ≈ 10 mg mL^−1^ NPs) were injected (100 µL) via the vena jugularis after anesthesia with isoflurane inhalation and buprenorphine (0.1 mg kg^−1^) (*n* = 6 for each NP sample). Additionally, the free dye used to fluorescently label the particles (CF647) was injected (100 µL) in the same concentration contained in a particle sample (50 *μ* m). After 5 min, blood sample was collected via i.v. punction while mice were still under anesthesia. After 1 h of particle circulation mice were anaesthetized with ketamine/xylazine, the final blood sample was collected, and they were killed through perfusion fixation with 4% PFA. The kidneys were harvested and cut transversally. They were cryoprotected by incubation in phosphate buffer (0.1 m pH 7.4) supplemented with 18% sucrose and 1% PFA overnight. Afterward, they were frozen in liquid nitrogen‐cooled 2‐propanol (−40 °C) and embedded in Tissue Tek O.C.T. Compound (Sakura Finetek). Kidneys were cut into 5 µm cryosections using a CryoStar NX70 cryostat (Thermo Fisher Scientific) and transferred onto Superfrost plus glass slides. Cell nuclei were stained with DAPI (12.5 µg mL^−1^ in DPBS) prior to imaging using an Axiovert 200M (Zeiss) fluorescence microscope and Zen System Software 2017 (Zeiss). Images of the whole kidney were acquired using a 10× objective. For glomerular fluorescence quantification images were taken using a 40× objective (an average of 120 glomeruli per sample for *n* = 6 mice per NP sample) and analyzed using Fiji Software.^[^ ^66^ ^]^ For better visualization the lookup table “Red Hot” was applied to the particle‐associated fluorescence. The area of each glomerulus was quantified, and the fluorescent area gated. Then, the integrated fluorescence density of each gated area was quantified and correlated to the whole glomerulus area. In order to compare the particle‐associated fluorescence of the inner and outer cortex, the cortex was divided into two equal sections and the glomerular fluorescence analyzed as described above.

##### Immunohistochemistry

To assess the glomerular localization of NPs, 5 µm kidney cryosections were washed for 5 min consecutively with DPBS, DPBS supplemented with 0.1% sodium dodecyl sulfate, and DPBS prior to 10 min‐blockage with 5% BSA in DPBS supplemented with 0.04% Triton‐X (m/v). Sections were washed again with DPBS (5 min) and incubated overnight with the primary polyclonal goat anti‐Integrin‐*α*8 antibody (1:200 in DPBS with 0.5% BSA and 0.004 Triton‐X [m/v]) at 4 °C. Then, they were washed for 5 min in DPBS and incubated for 1 h with the Cy2‐anti‐goat secondary antibody (1:400) and DAPI (12.5 µg mL^−1^) in DPBS supplemented with 0.5% BSA and 0.04% Triton‐X at room temperature light protected. Cryosections were washed with DPBS and ultrapure water before they were mounted using Dako Faramount Mounting Medium (Agilent Technologies) and analyzed using a Zeiss LSM 700 microscope and Fiji software, as described above.

##### Statistics

Statistical analysis was performed using GraphPad Prism Software 6.0. Student's *t*‐test (Figures 3C, 5A/C,D, and 7B) or two‐way ANOVA with a Sidak's (Figures 3D, 5B, and 7C) or Tukey's (Figure 3F) multiple comparison test were employed to evaluate statistical significance. Levels of statistical significance and “*n*” numbers for each experiment are indicated in the text and figure legends.

## Conflict of Interest

The authors declare no conflict of interest.

## Supporting information

Supporting Information
