# Supplementary material for: Nanoparticles Mimicking Viral Cell Recognition Strategies Are Superior Transporters into Mesangial Cells
Source: Adv Sci (Weinh). 2020 Apr 22;7(11):1903204. doi: 10.1002/advs.201903204 (PMC7284201; doi:10.1002/advs.201903204)
Supplement: Supplementary file 1 — Supporting Information [file ADVS-7-1903204-s001.pdf]

Copyright WILEY-VCH Verlag GmbH & Co. KGaA, 69469 Weinheim, Germany, 2020.

Supporting Information

Nanoparticles Mimicking Viral Cell Recognition Strategies are Superior Transporters into Mesangial Cells

*Sara Maslanka Figueroa, Daniel Fleischmann, Sebastian Beck, Philipp Tauber, Ralph Witzgall, Frank Schweda, and Achim Goepferich**

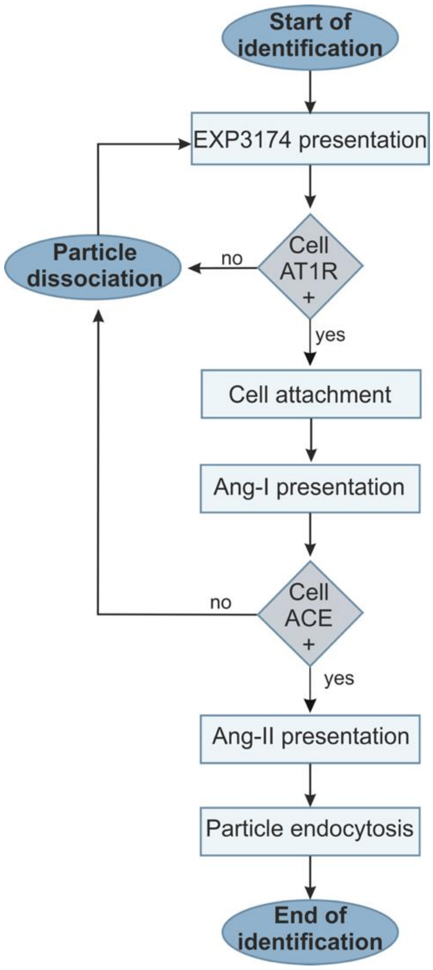

Figure S1. **Flow chart.** Exemplification of the triple target cell recognition of virus-mimetic NPs.

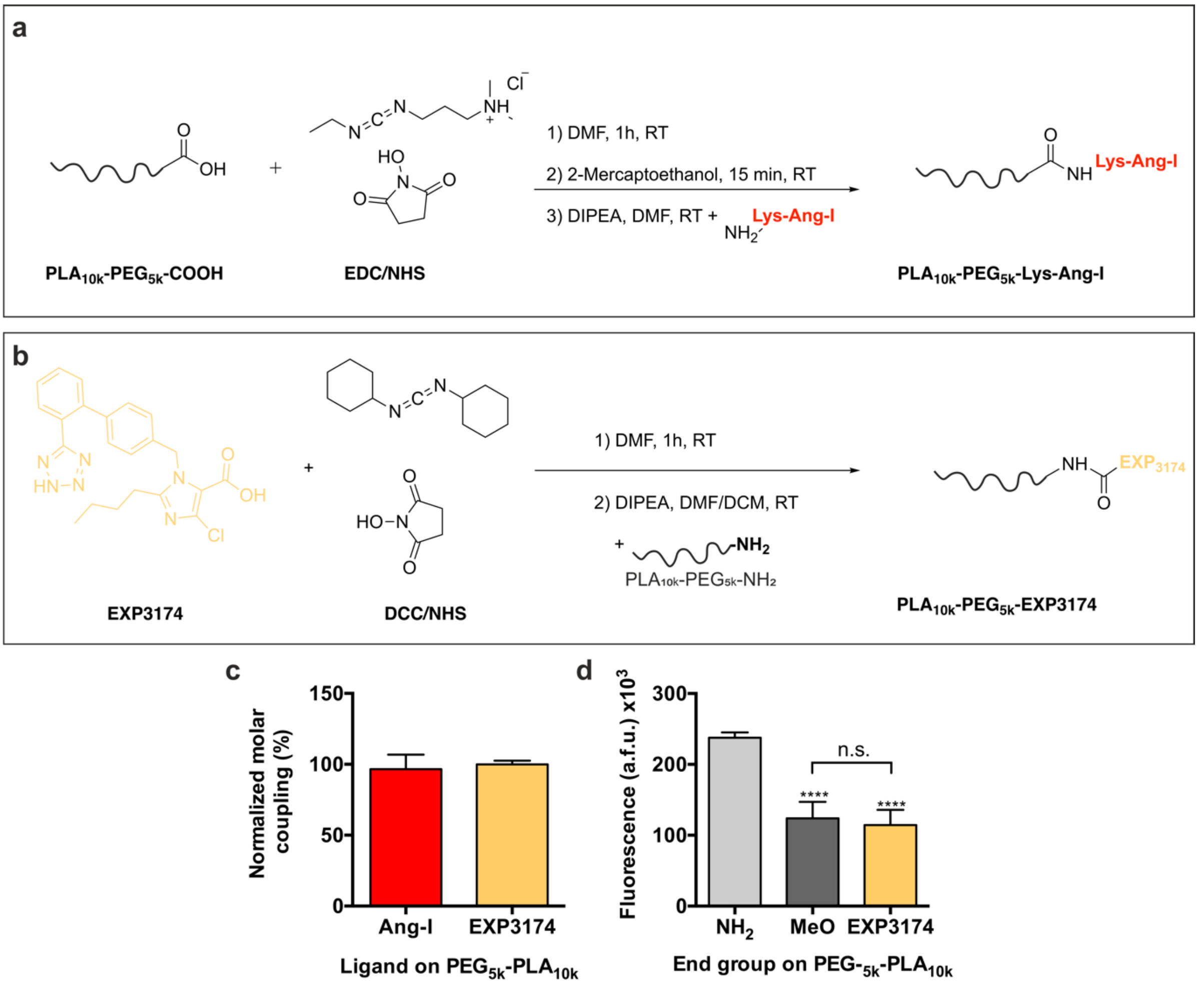

Figure S2. **Ligand coupling to PEG-PLA block copolymers.** a) Lys-Ang-I and b) EXP3174 were linked to carboxylic acid- or amine- ended PEG_{5k}-PLA_{10k} using EDC/NHS or DCC/NHS chemistry, respectively. c) Complete polymer functionalization shown by the quantification of the molar ligand and PEG content. For that, polymers were solubilized in acetonitrile at a concentration of 40 mg mL^{-1} and precipitated in stirring ultrapure water to create polymer micelles (final concentration 1 mg mL^{-1}). Coupled Ang-I was quantified using a Pierce BCA assay kit after the manufacturer’s instructions using a FLUOstar Omega microplate reader (BMG Labtech). EXP3174 was fluorescently quantified at λ_{ex}=250 nm and λ_{em}=370 nm using a LS-5S fluorescence plate reader (PerkinElmer). d) Absence of unreacted NH_2 polymer end groups on EXP3174-modified-polymer was determined using flurescamine.[1] A Student´s t-test was performed using GraphPad Prism 6.0 to assess

statistical significance. Levels of statistical significance ae indicated as ****p $\leq$ 0.0001 comparing the fluorescence of MeO- and EXP3174- with NH_2-terminated PEG_{5k}-PLA_{10k}.

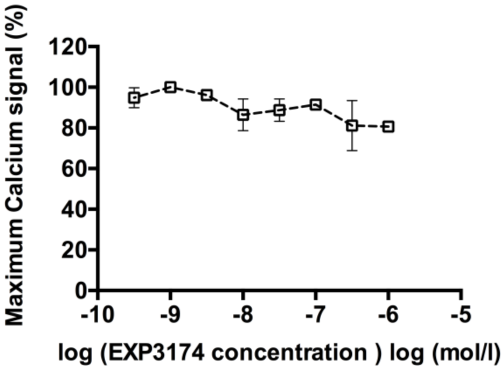

Figure S3. **Maximum calcium signal and inhibition by NPEXP.** rMCs (90,000 cells per well) were stimulated simultaneously with Ang-II (400 n_M) and NPEXP and the resulting intracellular calcium response measured immediately for 1 minute. At the used NPEXP concentrations, the EXP3174 ligand did not inhibit the agonist-triggered calcium signal during the assay duration. Therefore, the influence of EXP3174 on the Ang-II-measurement was considered negligible. Results are shown as mean ± SD of at least n = 3 measurements.

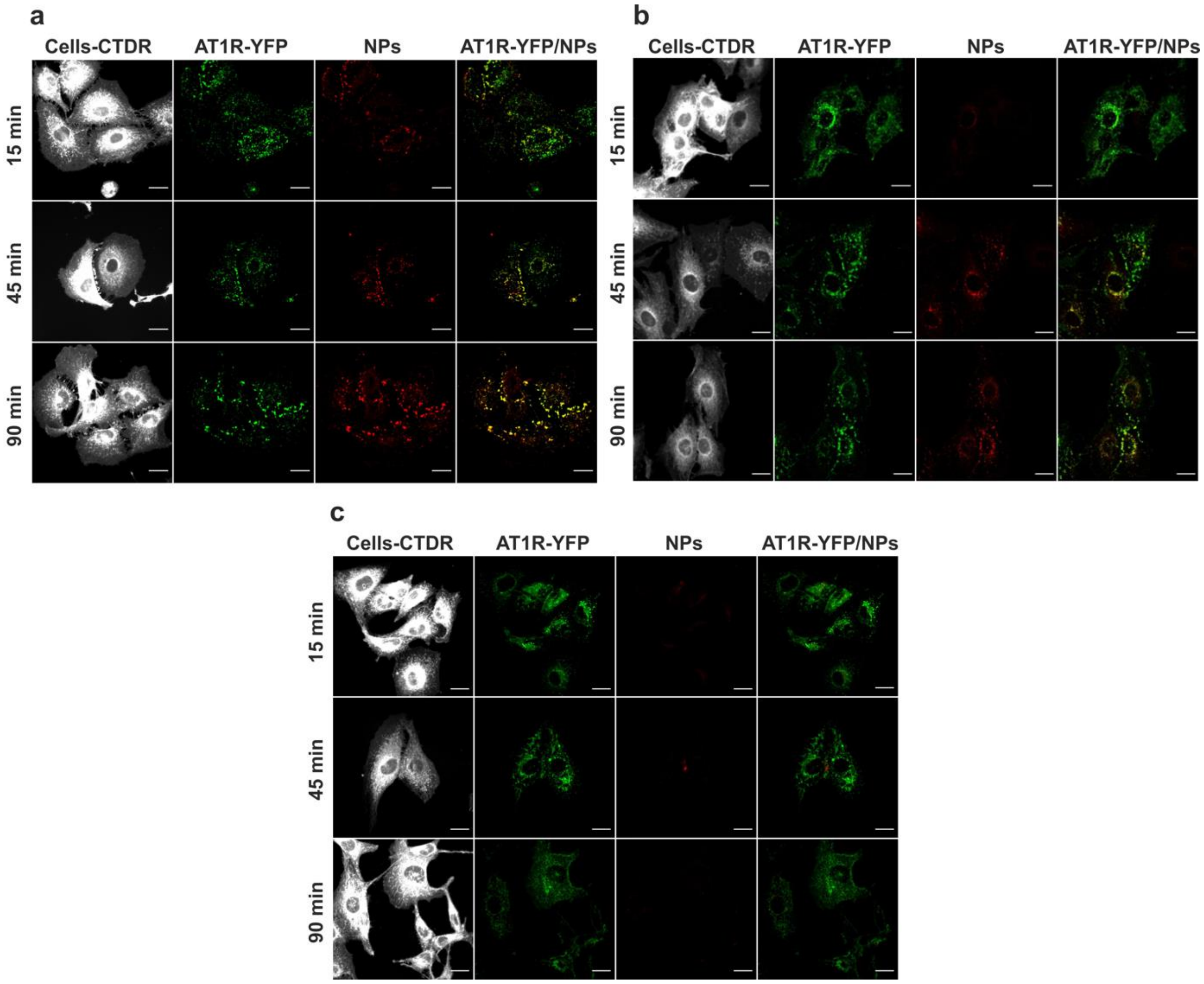

Figure S4. Uptake of different particle formulations over time in AT1R positive pAT1R-rMCs analysed through CLSM. a) NPEXP are not internalized in the cell line and mostly locate on the cellular membrane and filipodia between cells forming big clusters over time. Receptor binding is shown by the colocalization of NP- and receptor- associated fluorescence. b) NPAng-I are internalized by the cells as depicted by their cytoplasmic localization. c) NPMeO do not associate with cells due to their lack of a tethered ligands enabling a specific targeting. White: cells; Green:AT1R-YFP; Red: NP-formulations. Scale bar 20 μm.

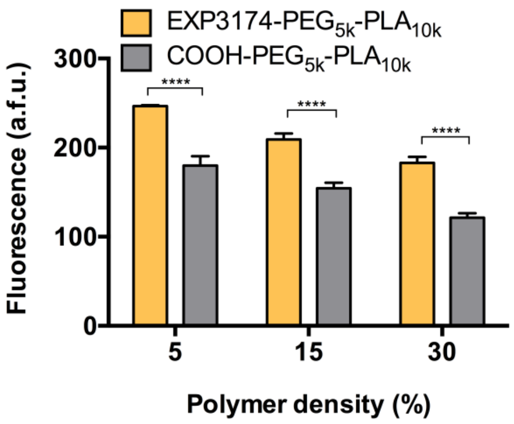

Figure S5. EXP3174 counterbalances the uptake decrease due to stearic hindrance of the Ang-I ligand by long polymer chains on NPEXPAng-I. NPAng-I (grey) with 20% Ang-I density were prepared with varying polymer densities of COOH-PEG$_{5k}$-PLA10k and analysed for their cellular uptake using flow cytometry. Concomitantly, NPEXPAng-I (yellow) were prepared with varying densities of EXP3174-PEG$_{5k}$-PLA10k to compare the effect of the second ligand on the stearic hindrance of Ang-I. Functionalization of long polymer chains with EXP3174 on NPEXPAng-I counterbalanced the decreased uptake due to stearic hindrance of the Ang-I ligand when adding non-functionalized long polymers, and significantly increased the particle internalization. Results are presented as mean ± SD of at least n = 3 measurements. A 2-way ANOVA with Sidak´s multiple comparisons test was performed using GraphPad Prism 6.0 to assess statistical significance. Levels of statistical significance ae indicated as ****$p \leq 0.0001$

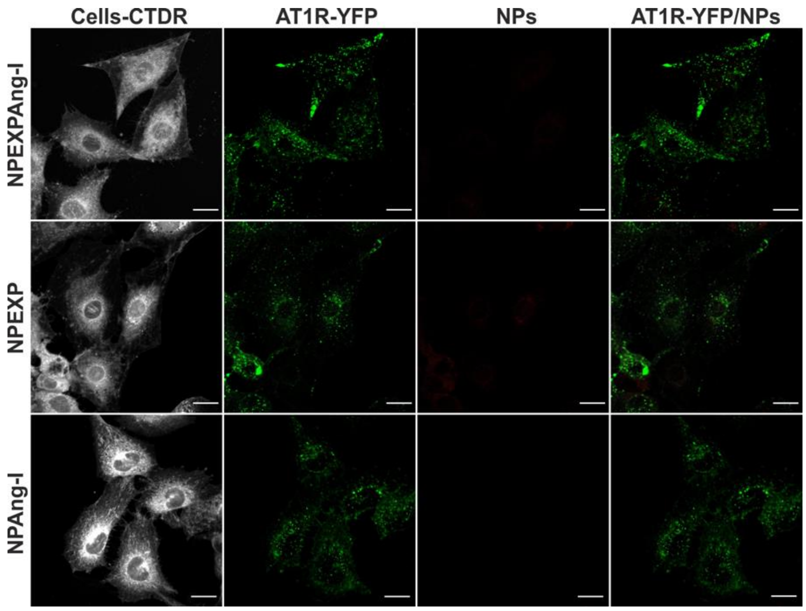

Figure S6. Specificity of the NP uptake analysed through CLSM. Cells were preincubated for 30 minutes with free EXP3174 prior to the addition of the different NP formulations (NPEXPAng-I, NPAng-I and NPEXP). Inhibition of the target receptor resulted in the suppression of the particle-associated fluorescence. Scale bar 20 μm.

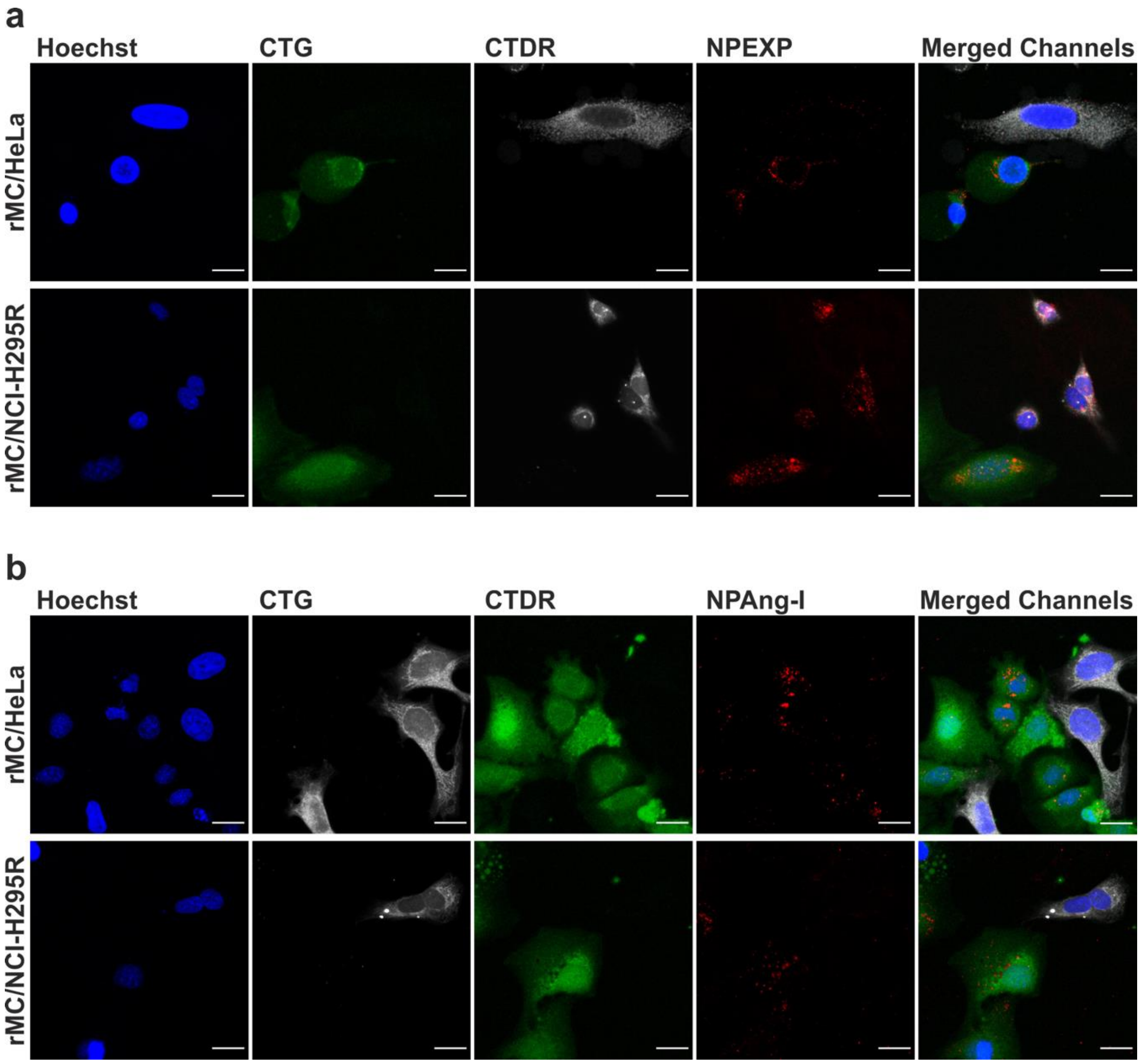

Figure S7. Uptake of a) NPEXP and b) NPAng-I in co-culture of target and off-target cells. NPEXP show accumulation in rMCs and NCI-H205R cells, as they both carry the AT1R. Contrary, the co-culture of rMCs and HeLa cells shows preferential accumulation of NPEXP in rMCs, as HeLa cells express only minor amounts of the receptor on the cell membrane. NPAng-I show a higher specificity as they accumulate in target rMCs, which carry the necessary equipment for their internalization (the ACE and the AT1R), over off-target cells lacking ACE (HeLa or NCI-H295R cells). Blue: cell nuclei; White: off-target cells (HeLa or NCI-H295R); Green: target cells (rMCs); Red: NPs. Scale bar 20 μm.

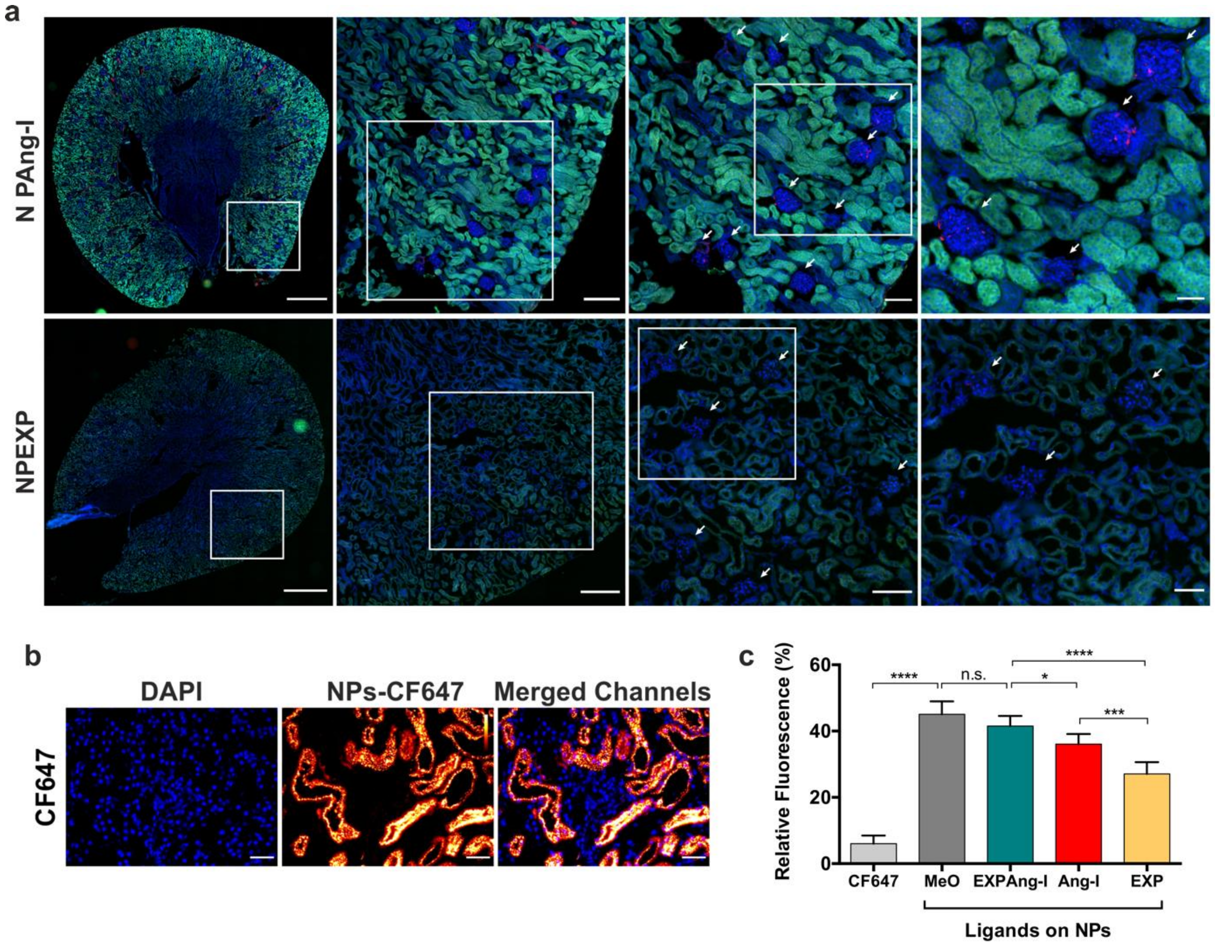

Figure S8. ***In vivo*** **distribution of different NP formulations.** a) Distribution of NPAng-I and NPEXP in mice kidneys**.** NPAng-I show low NP-associated fluorescence in the majority of glomeruli (white arrows) contrary to NPEXP which did not accumulate in this area. From left to right squared out regions are shown as magnifications. Scale bar from left to right 900, 200, 100 and 50 μm. Blue: DAPI staining of cell nuclei; Green: tissue autofluorescence; Red: NPs. b) Kidney distribution of the free CF647 dye used to label the NPs. Strong fluorescence could be detected in the tubular area, with no fluorescence in the glomeruli (white circle), as due to its low molecular size it can be freely filtrated. c) Plasma residence of different NP-formulations after 1-hour circulation in NRMI mice normalized to the fluorescence measured 5 minutes after injection. NP-associated fluorescence in plasma was measured using a FLUOstar Omega microplate reader (BMG Labtech) with excitation and emission wavelengths of 640 and 680 nm, respectively. Fluorescence 1 hour after injection was

correlated to the initial fluorescence 5 minutes after injection. Non-targeted NPs (NPMeO) show the highest blood circulation time due to the stealth effect conferred by the PEG-shell. This is matched by NPEXPAng-I even though ligands cover 40% of the NP surface, which usually decreases a particles stealth effect. They depict a significant higher fluorescence in plasma after 1 h compared to particles functionalized with only one ligand (NPAng-I and NPEXP). NPAng-I, which carry a specific two-step virus-mimetic recognition mechanism also show a significant superior blood residence than NPEXP, which represent commonly targeted NPs. As a control, the free dye used to label the particles (CF647) was injected into mice, which rapidly disappears from the blood circulation due to its free filtration. Results in c) are presented as mean ± SD of at least n = 6 samples. A Student t test was performed using GraphPad Prism 6.0 to assess statistical significance. Levels of statistical significance are indicated as *p ≤ 0.05, ***p ≤ 0.001 and ****p ≤ 0.0001. n.s.: non-significant.

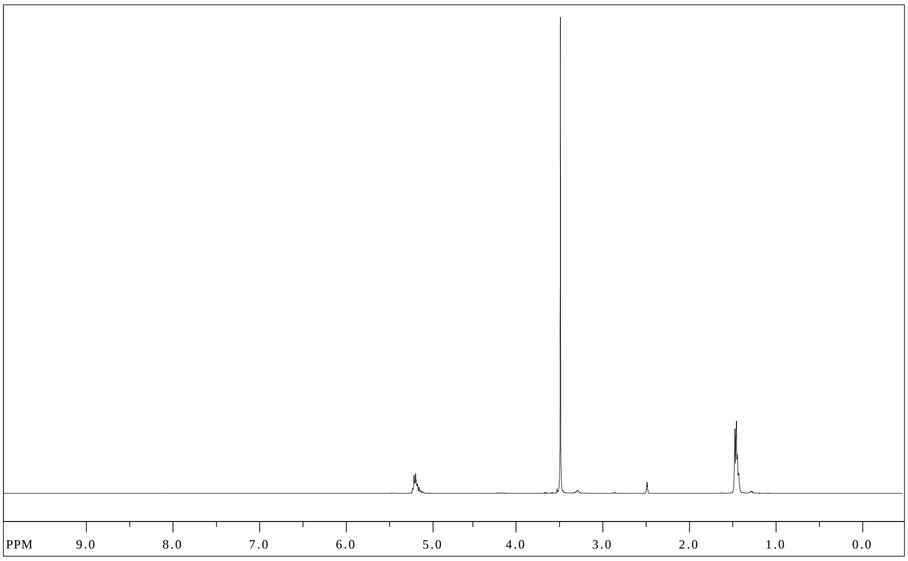

Figure S9. ^{1}H-NMR (DMSO-d6, 400 MHz) spectra of NH_2-PEG_{5k}-PLA_{010k}. δ (ppm): 1.44 ppm (-C(CH3)H-), 2.50 ppm (solvent peak), 3.30 ppm (H3COCH2CH2-), 3.49 ppm (-OCH2CH2-), 4.21 ppm (-OCH2CH2-O(CO)-), 5.17 ppm (-C(CH3)H-).

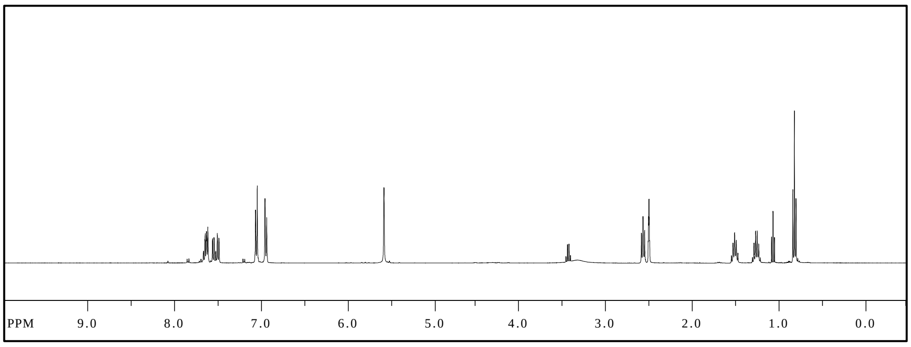

Figure S10. ^{1}H-NMR (DMSO, 400 MHz) spectra of EXP3174. δ (ppm): 0.79 ppm (-CH3CH2), 1.25 (CH3CH2CH2), 1.48 (CH2CH2CH2), 2.50 (solvent peak), 2.56 ppm (CH2CH2CN2), 5.45 ppm (NCH2C(CH)CH), 6.93 ppm (=CHC(H)=CH), 7.05 ppm ((=CHC(H)=CH), 7.51 ppm ((=CHC(H)=CH), 7.98 ppm (=CHC(H)=C).

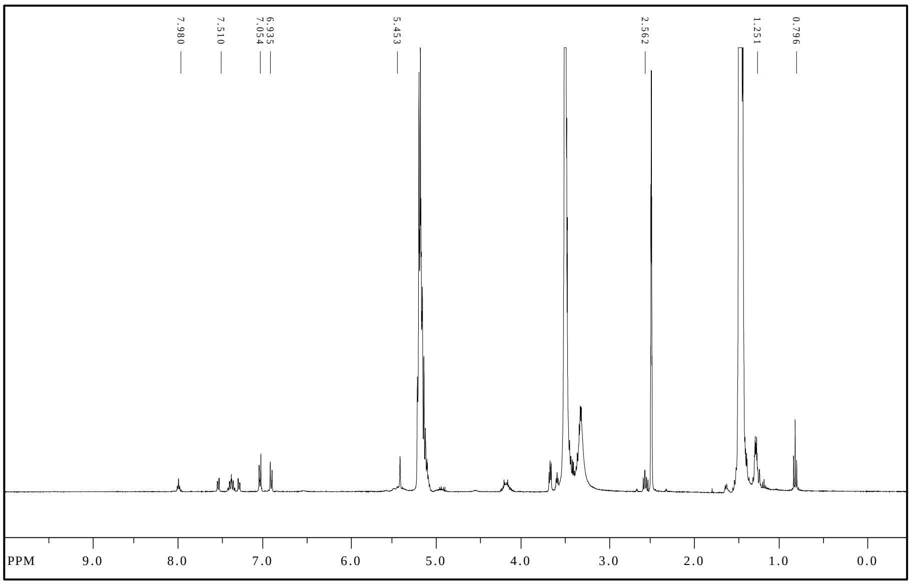

Figure S11. ^{1}H-NMR (DMSO,400 MHz) spectra of EXP3174-PEG$_{5k}$-PLA$_{10k}$. Characteristic EXP3174 shifts (marked) confirm the successful coupling of EXP3174 to NH$_2$-PEG$_{5k}$-PLA$_{10k}$. δ (ppm): 0.79 ppm (-H3C-CH2), 1.25 (H3C-CH2-CH2), 1.44 ppm (-C(CH3)H-), 1.48 (CH2CH2CH2), 2.50 (solvent peak), 2.56 ppm (CH2CH2CN2), 3.30 ppm (H3COCH2CH2-), 3.49 ppm (-OCH2CH2-), 4.21 ppm (-OCH2CH2-O(CO)-), 5.17 ppm (-C(CH3)H-), 5.57 ppm (NCH2C(CH)CH), 6.93 ppm (=CHC(H)=CH), 7.05 ppm ((=CHC(H)=CH), 7.54 ppm ((=CHC(H)=CH), 7.65 ppm (=CHC(H)=C).

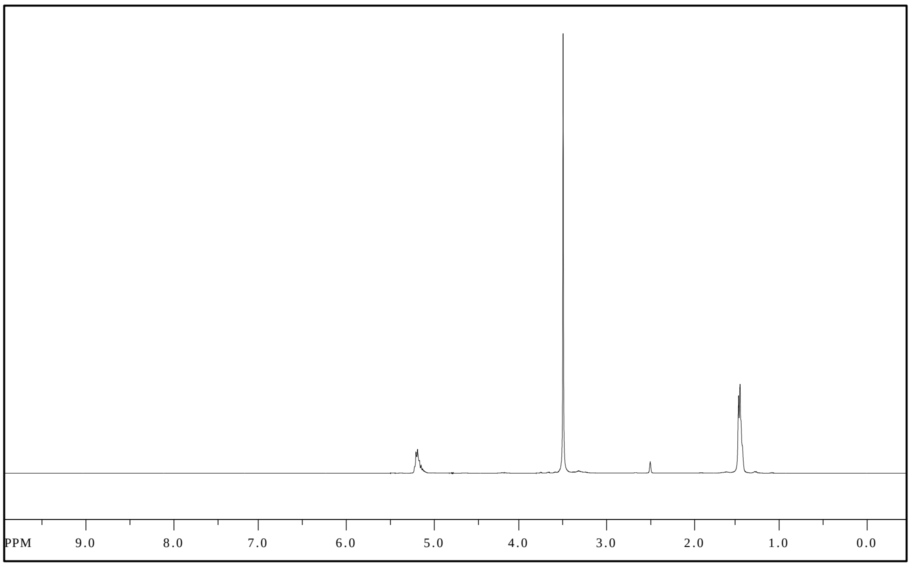

Figure S12. ^{1}H-NMR (DMSO,400 MHz) spectra of COOH-PEG_{5k}-PLA_{10k}. δ (ppm): 1.46 ppm (-C(CH3)H-), 2.50 ppm (solvent peak), 3.30 ppm (H3COCH2CH2-), 3.49 ppm (-OCH2CH2-), 4.19 ppm (-OCH2CH2-O(CO)-), 5.19 ppm (-C(CH3)H-).

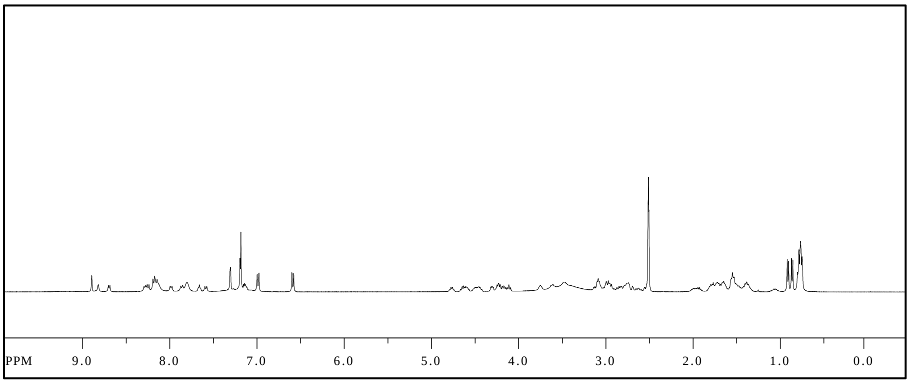

Figure S13. ^{1}H-NMR (DMSO-d6, 400 MHz) spectra of Lys-Ang-I. δ (ppm): 0.73 (-CH(CH3)CH3), 0.84 (CH3-CH(CH3)CHN), 2.50 (solvent peak), 6.59 ppm (=CHC(H)=CH), 7.20 ppm (=CHC(H)=CH), 8.20 ppm CO(NH)CH(CH2)CO.

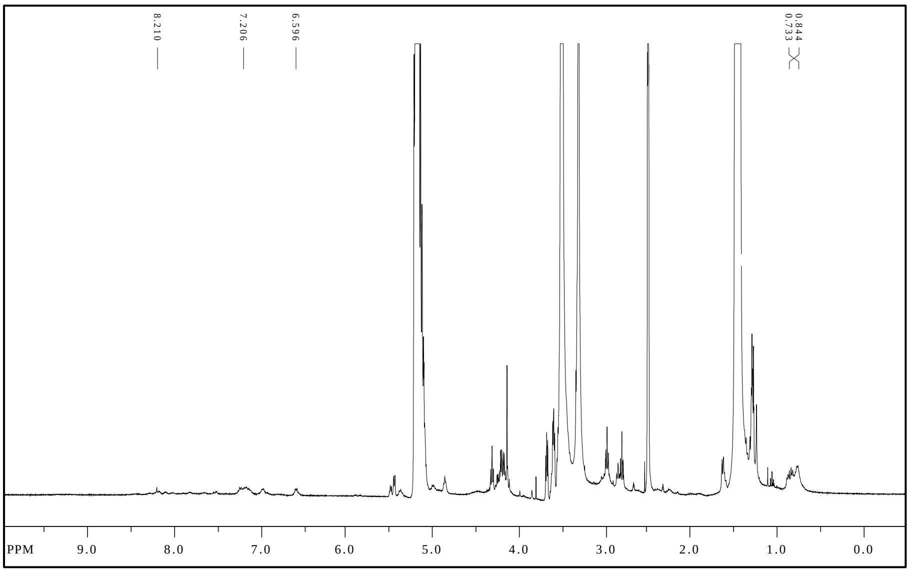

Figure S14. ^{1}H-NMR (DMSO-d6, 400 MHz) spectra of Ang-I- PEG_{5k}-$PLA_{10k.}$ Characteristic Lys-Ang-I shifts (marked) confirm the successful coupling of Lys-Ang-I to COOH-PEG_{5k}-PLA_{10k}. δ (ppm): 0.73 (-CH(CH3)CH3), 0.84 (CH3-CH(CH3)CHN), 2.50 (solvent peak), 6.59 ppm (=CHC(H)=CH), 7.20 ppm (=CHC(H)=CH), 8.20 ppm CO(NH)CH(CH2)CO.

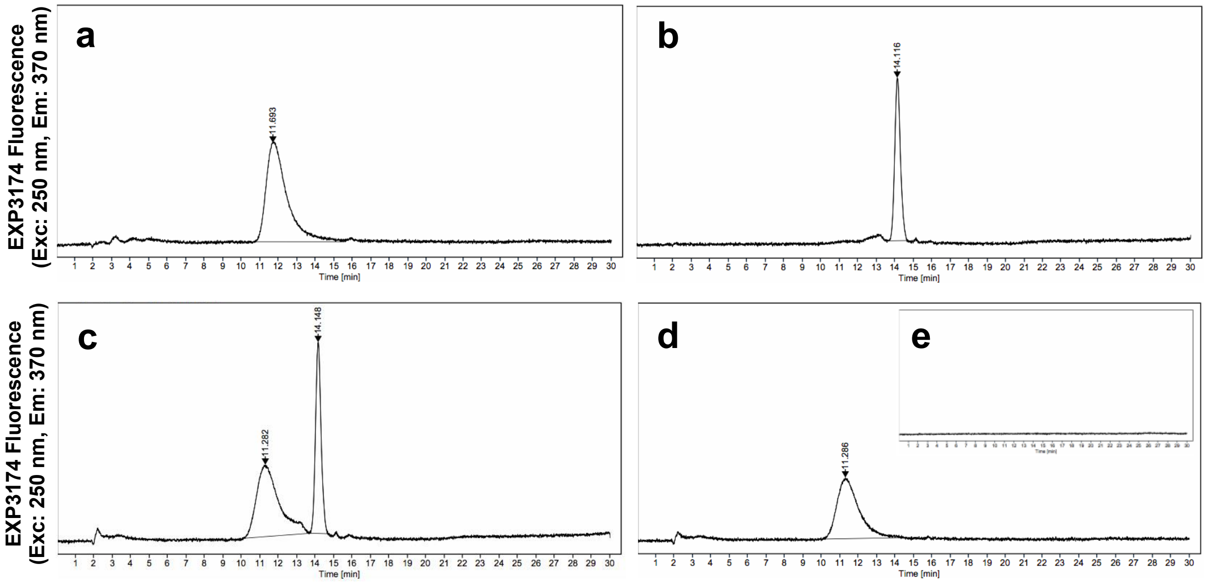

Figure S15. Coupling confirmation of EXP3174 to block copolymer PEG_{5k}-PLA_{10k}. An Agilent PLRP-S 4000A 8 µm 150x4.6 mm column and an Agilent Infinity 1260 HPLC were used. Elution was obtained by using the following gradient of solvents A (Water/Acetonitrile (95/5) (v/v) with 0.1% TFA) and B (Acetonitrile/Water (95/5) (v/v) with 0.085% TFA): 75/25 (A/B) to 72/25 (A/B) in 5 minutes to 15/85 (A/B) in 30 minutes. Samples (10 µL) were injected at a concentration of 10 µM. The column was operated at 40 ºC and a flow rate of 1 mL/min. EXP3174 fluorescence was excited at 250 nm and detected at 370 nm. HPLC chromatograms of a) free EXP3174, b) EXP-PEG_{5k}-PLA_{10k} c), EXP-PEG_{5k}-PLA_{10k} mixed with free EXP3174, d) COOH-PEG_{5k}-PLA_{10k} mixed with free EXP3174, and e) COOH-PEG_{5k}-PLA_{10k}. b) shows the absence of free EXP3174, proving the success of the purification procedure.

REFERENCES

[1] R. E. Smith, R. MacQuarrie, *Anal. Biochem.* **1978**, *90*, 246.
